# ID 149 - Ezetimiba para o Tratamento de Indivíduos com Alto a Muito Alto Risco Cardiovascular: análise de custo-efetividade e impacto orçamentário

**DOI:** 10.5327/2237-9622.2025.v34s1.149

**Published:** 2025-11-25

**Authors:** Layssa Andrade Oliveira, Mariana Millan Fachi, Juliana Yukari Viscondi, Tassiane Cristine Santos de Paula, Haliton Alves Oliveira, Rosa Camila Lucchetta

**Keywords:** ezetimiba, estatinas, doenças cardiovasculares, fatores de risco de doenças cardíacas

## Abstract

**Introdução:**

As doenças cardiovasculares (DCV) são uma das principais causas de morbimortalidade global. As estatinas são a abordagem terapêutica de primeira linha para DCV em pacientes com níveis aumentados de colesterol ou com risco aumentado de DCV, visando reduzir o colesterol LDL. Mas nem todos os pacientes atingem as metas desejadas apenas com as estatinas ou toleram essa medicação em altas doses. Nesse contexto, a ezetimiba ao reduzir a absorção intestinal de colesterol pode ser uma alternativa útil quando combinada às estatinas. Sendo assim, o objetivo deste estudo foi avaliar a custo-efetividade e o impacto orçamentário da ezetimiba no tratamento de pacientes com alto risco cardiovascular para o Sistema Único de Saúde (SUS).

**Método:**

Foi realizada uma avaliação econômica (AE) para estimar a relação de custo-efetividade incremental da ezetimiba combinada com estatinas (intervenção) em comparação a estatinas isoladas (comparador). Utilizou-se um modelo de Markov com horizonte temporal lifetime (correspondendo a xx anos) e desfechos de anos de vida ajustados pela qualidade (QALY) e anos de vida (AV), bem como custos diretos médicos com tratamento, exames e monitoramento. Além disso, uma taxa de desconto de 5% foi aplicada para custos e desfechos. Para análise de impacto orçamentário foram estimados o número de pacientes elegíveis por demanda aferida em um horizonte temporal de cinco anos, além de ter sido estimada uma taxa de difusão da tecnologia de 10% ao ano, chegando em 50% no quinto ano. O modelo levou em conta apenas os custos das tecnologias em avaliação.

**Resultados:**

A análise demostrou um benefício clínico adicional de 0,0842 QALY e 0,1020 AV com a intervenção, com um custo incremental de R$ 858,42. As razões de custo-efetividade incrementais foram R$ 10.192,63/QALY ganho e R$ 8.415,83/AV ganho, com uma probabilidade de 73% de ser custo-efetiva dentro dos limiares recomendados. No impacto orçamentário, considerando uma população elegível variando de 147.565 a 150.793, a incorporação da ezetimiba pode gerar incremento de custos variando de R$ 1.142.661 no primeiro ano a R$ 5.838.310 no quinto ano, totalizando cerca de R$ 17,4 milhões acumulados em cinco anos. Variações nos preços dos medicamentos podem elevar o impacto orçamentário acima da estimativa inicial, ou até mesmo gerar economia ao sistema, conforme demonstrado nas análises de sensibilidade.

**Conclusão:**

A análise de custo-efetividade mostrou que a intervenção investigada oferece maior benefício clínico e apresenta um custo total maior em comparação ao tratamento padrão. No entanto, é considerada custo-efetiva para os limiares adotados pelo SUS (R$ 40.000/QALY ganho e R$ 35.000 por AV/ganho). A análise de impacto orçamentário sugere que a incorporação da ezetimiba pode resultar em incremento de custo ao sistema, mas que apresenta importante incerteza a depender do preço das tecnologias avaliadas.

